# Essential role for ALCAM gene silencing in megakaryocytic differentiation of K562 cells

**DOI:** 10.1186/1471-2199-11-91

**Published:** 2010-12-02

**Authors:** Fang Tan, Samit Ghosh, Flaubert Mbeunkui, Robert Thomas, Joshua A Weiner, Solomon F Ofori-Acquah

**Affiliations:** 1 Department of Pediatrics, Aflac Cancer Center and Blood Disorders Service, Emory University School of Medicine, Atlanta, GA 30322, USA; 2 Department of Cell Biology and Neuroscience, University of South Alabama, Mobile, AL 36688, USA; 3 Department of Biology, The University of Iowa, Iowa City, IA 52242, USA

## Abstract

**Background:**

Activated leukocyte cell adhesion molecule (ALCAM/CD166) is expressed by hematopoietic stem cells. However, its role in hematopoietic differentiation has not previously been defined.

**Results:**

In this study, we show that ALCAM expression is silenced in erythromegakaryocytic progenitor cell lines. In agreement with this finding, the ALCAM promoter is occupied by GATA-1 *in vivo*, and a cognate motif at -850 inhibited promoter activity in K562 and MEG-01 cells. Gain-of-function studies showed that ALCAM clusters K562 cells in a process that requires PKC. Induction of megakaryocytic differentiation in K562 clones expressing ALCAM activated PKC-δ and triggered apoptosis.

**Conclusions:**

There is a lineage-specific silencing of ALCAM in bi-potential erythromegakaryocytic progenitor cell lines. Marked apoptosis of ALCAM-expressing K562 clones treated with PMA suggests that aberrant ALCAM expression in erythromegakaryocytic progenitors may contribute to megakaryocytopenia.

## Background

Hematopoiesis is controlled by interactions between hematopoietic stem cells and their microenvironment. These interactions influence retention of stem cells in specific niches, and stem and progenitor cell expansion, lineage divergence and differentiation [1]. Adhesion molecules are major regulators of cell-cell interactions and they influence multiple aspects of hematopoiesis [1-4]. Indeed, antibodies against various adhesion molecules including VLA-4 and VCAM-1 inhibit the ability of hematopoietic stem cells to populate the bone marrow of irradiated mice [5], and gene knock-out studies of integrins have shown their critical role in homing and colonization of late-stage primary hematopoietic organs such as the embryonic liver [6,7]. More recently, N-cadherin expression has been implicated in retention of hematopoietic stem cells in the bone marrow niche [8-10] although this claim is not supported by other studies [11]. In contrast to their role in homing, our understanding of adhesion molecule biology in lineage commitment and differentiation is poorly defined.

Hematopoietic cell antigen, also known as activated leukocyte cell adhesion molecule (ALCAM/CD166), is a member of the immunoglobulin super-family. It is expressed on the surface of the most primitive hematopoietic cells in human fetal liver and fetal and adult bone marrow [12]. Other studies have found ALCAM expression on subsets of stromal cells in the para-aortic mesoderm and other primary sites of hematopoiesis in the human embryo [13]. ALCAM-mediated interactions are important during neural development [14], maturation of hematopoietic stem cells in blood forming tissues [12,15], immune responses [16] and in tumor progression [17]. Anti-ALCAM antibodies inhibit myeloid colony formation *in vitro *by mechanism that remains unknown [18]. We showed previously that ALCAM is involved in transmigration of monocytes across endothelial monolayers [19]. More recent *in vivo *studies have shown that ALCAM is essential for monocyte migration across the blood-brain barrier [20]. Other studies indicate the interaction of ALCAM on dendritic cells with the T-cell ligand CD6 is required for optimal T-cell activation [21]. While these studies highlight ALCAM's role in mature and activated leukocyte cell biology, there is currently no information on ALCAM's role in hematopoietic progenitor cell biology.

In this study, we examined ALCAM expression in human hematopoietic cell lines. The ALCAM gene was cloned and functionally characterized in K562 cell lines. The influence of ALCAM on megakaryocytic differentiation of K562 cells was investigated.

## Results

### Lineage-specific expression of ALCAM in hematopoietic progenitor cell lines

**Figure 1:**
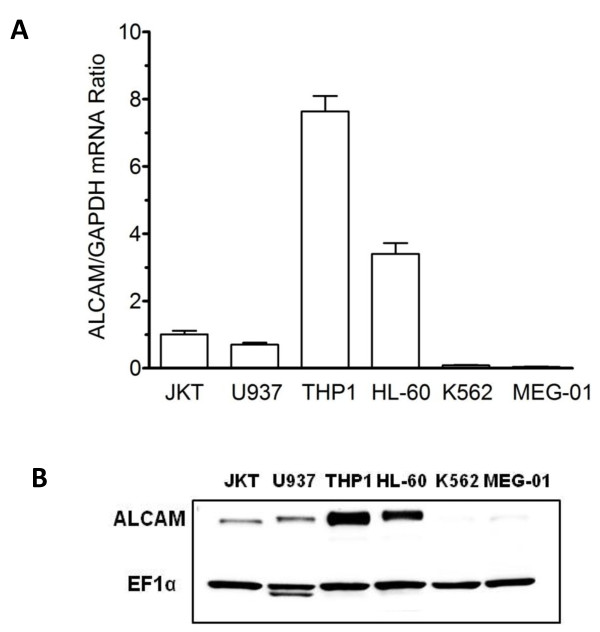
**ALCAM expression in hematopoietic progenitor cell lines**. A) Total RNA was isolated from hematopoietic cells and ALCAM mRNA quantified by quantitative RT-PCR. GAPDH was used as invariant control in the experiment. Data shown is the mean of three analyses each in triplicates. B) Whole cell lysates (15 μg) from indicated cells were blotted for ALCAM protein by western blot analysis and protein loading verified by analyzing the same filters for EF-1α expression.

Previous studies have documented ALCAM surface expression on hematopoietic stem and progenitor cells. In this study, we quantified ALCAM mRNA expression in multiple human hematopoietic progenitor cell lines of myeloid, lymphoid, erythroid, and megakaryocytic lineages by real-time quantitative PCR. ALCAM mRNA was most abundant in THP-1 monocytes, at a level 2-fold higher than in HL-60 cells, and 8-fold higher than in U-937 and Jurkat cells (Figure 1A). No ALCAM transcripts were however detected in K562 and MEG-01 cells (Figure 1A). This expression pattern was confirmed at the protein level as none of the erythromegakaryocytic progenitor cell lines (K562, MEG-01) expressed ALCAM, while ALCAM protein was abundant in THP-1 monocytes (Figure 1B).

### A negative GATA-1 binding element in the ALCAM promoter

**Figure 2:**
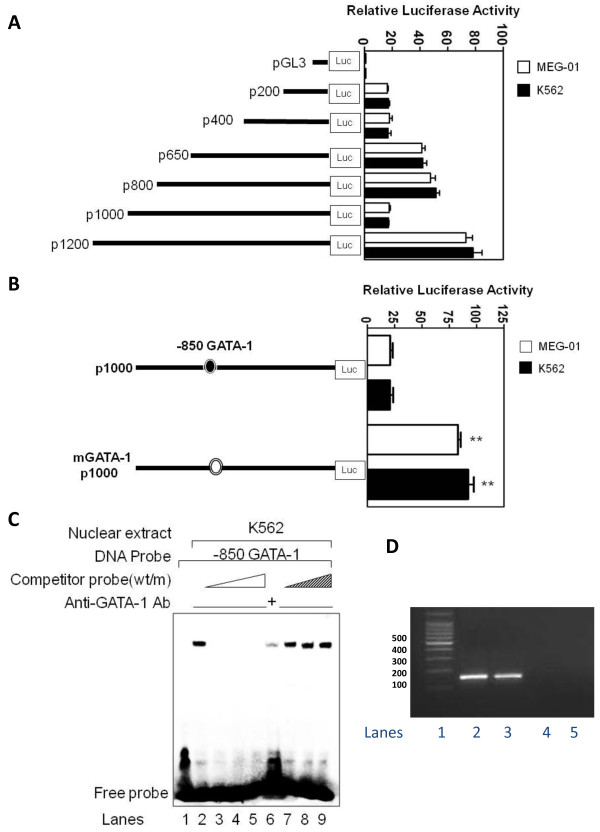
**A functional GATA-1 element in the proximal ALCAM promoter**. A) Schematic diagram of reporter constructs consisting of truncated ALCAM promoter fragments driving expression of pGL3. Histogram shows the relative luciferase activity for each construct in K562 and MEG-01 cells. B) Reporter constructs showing the presence of wild-type (filled circle) or mutant (open circle) GATA-1 *cis *element at -850 in the human ALCAM promoter. Histogram shows the relative luciferase activity for each construct in K562 and MEG-01 cells. C) Electrophoretic mobility shift assay showing protein-DNA complexes in experiments performed with the -850 GATA probe and no nuclear extracts (lane 1), or with K562 nuclear extracts alone (lane 2), and in the presence of molar excess of unlabeled wild-type probe (lanes 3-5), anti-GATA1 antibody (lane 6) and unlabeled mutant -850 GATA1 probe (lanes 7-9). D) Ethidium bromide stained gel of ChIP assay PCR products showing a 151 bp product for input DNA (lane 2), and products from chromatin immunoprecipitations performed with anti-GATA1 antibody (lane 3), IgG (lane 4) and without chromatin (lane 5). Lane 1 contains DNA size marker.

Thus far, we had identified an expression pattern for ALCAM that was consistent with regulation of the ALCAM gene by erythroid and megakaryocytic transcription factors. To investigate this idea, multiple fragments of the ALCAM 5'-flanking region was cloned, sequenced and its activity analyzed in K562 and MEG-01 cells. Activity of the p650 construct was on average 40-fold higher compared to pGL3 in both cell types (Figure 2A). Activity of p1000 was significantly lower compared to p800, which suggested the presence of negative regulatory *cis *element in the interval -800 to -1000 of the ALCAM gene. Sequence analysis using the TRANSFAC 7.0 software identified a GATA-1 motif at -850. Mutation of this *cis *element increased activity of p1000 by 3-fold (Figure 2B). To confirm a role for GATA-1, K562 nuclear extracts were used in gel mobility shift assay. A single major protein-DNA complex formed on a -850 ALCAM DNA probe using nuclear extracts from K562 and MEG-01 cells (Figure 2C, lane 2 and data not shown). Formation of this complex was competitively blocked by unlabeled wild-type -850 ALCAM GATA probe indicating the specificity of this *cis *element in the protein-DNA complex (lanes 3-5), and by anti-GATA-1 antibody (lane 6), which confirmed that the GATA-1 protein is a component of this protein-DNA complex. On the contrary, increasing molar excess of an unlabeled mutant -850 ALCAM probe failed to compete for binding (lanes 7-9). Finally, we prepared chromatin from K562 cells to determine whether GATA-1 occupied the ALCAM promoter *in vivo*. Results of ChIP assay experiments confirmed that GATA-1 binds to the endogenous promoter in K562 cells (Figure 2D). Collectively, these findings suggest that ALCAM silencing is part of the mechanism of GATA-1 mediated megakaryocytic differentiation.

### Ectopic ALCAM expression clusters K562 cells in a PKC-dependant manner

**Figure 3:**
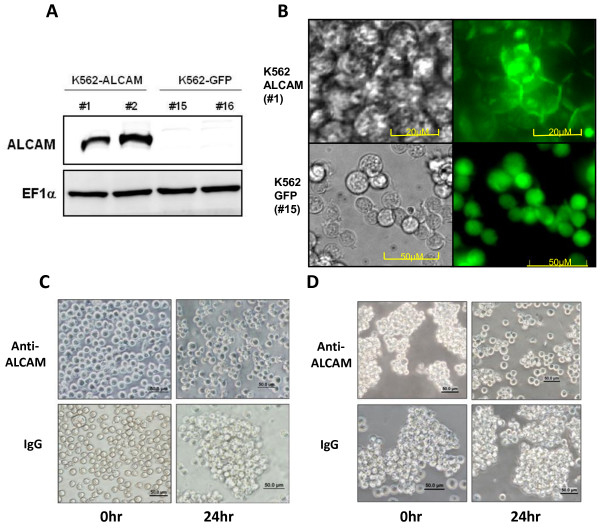
**Ectopic expression of ALCAM clusters K562 cells**. A) Western blot analysis shows expression of ALCAM in K562 clones stably transfected with ALCAM-GFP (#1, #2), but not in clones transfected with empty vector (#15, #16). B) Live-cell imaging of K562-ALCAM (#1) and control K562 (#15) clones. C) Photomicrographs of K562-ALCAM cells seeded with anti-ALCAM anti-body or IgG and cultured for 24 hours. Note formation of a large aggregate of cells in cultures containing IgG. D) Photomicrographs of pre-formed K562-ALCAM cell clusters treated with anti-ALCAM antibody or IgG for 24 hours. Anti-ALCAM antibody disaggregates cell clusters while IGg has no impact.

**Figure 4:**
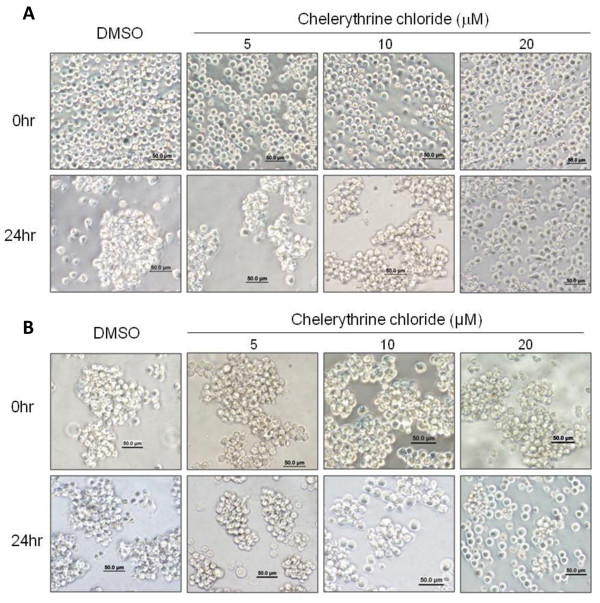
**ALCAM-induced clustering of K562 cells is PKC-dependant**. A) Photomicrographs of K562-ALCAM single cell suspensions treated with a concentration (5-20 μM) range of chelerythrine chloride or DMSO (vehicle) at the time of seeding (0 hr) and 24 hours later. Chelerythrine chloride dose-dependently inhibits the formation of clusters of K562-ALCAM. B) Photomicrographs of preformed K562-ALCAM clusters treated with a concentration (5-20 μM) range of chelerythrine chloride or DMSO (vehicle). Chelerythrine chloride dose-dependently disaggregates pre-formed clusters. Cultures were photographed using Olympus IX50 microscope (Olympus Optical Co. Ltd. Japan).

To determine the functional relevance of ALCAM silencing, we established clones of K562 stably expressing ALCAM linked to a green florescent protein (K562-ALCAM) or a GFP vector (K562). ALCAM was abundantly expressed in these stable K562 clones while it was absent in the control clones expressing GFP (Figure 3A). Live-cell imaging showed that ALCAM was recruited to sites of cell-cell contact in the K562-ALCAM clones whereas GFP remained distributed in the cytosol in the control K562-GFP clones (Figure 3B). Unlike parental K562 and K562-GFP clones, the K562-ALCAM clones consistently formed large clusters in culture, spontaneously. To test whether ALCAM is responsible for this behavior, a function blocking anti-ALCAM antibody that we and others have shown previously to inhibit ALCAM-mediated homotypic adhesion was added to cultures. Anti-ALCAM antibody (10 μg/ml) prevented cell clustering whereas an isotype-matched IgG had no effect (Figure 3C). Moreover, anti-ALCAM antibody disaggregated pre-formed K562-ALCAM clusters while isotype-matched IgG had no impact (Figure 3D). PKC-α has previously been implicated in ALCAM-mediated cytoskeletal alterations in K562 cells, therefore we tested whether this mediator was involved in ALCAM-induced K562 clustering. Inhibition of PKC dose-dependently blocked clustering of K562-ALCAM cells, while DMSO had no impact on this behavior (Figure 4A). Moreover, the pan-PKC inhibitor chelerythrine chloride dose-dependently disaggregated previously formed large aggregates of K562-ALCAM cells (Figure 4B). These data indicate that PKC contributes to the ALCAM-mediated aggregation of hematopoietic cells.

**Figure 5:**
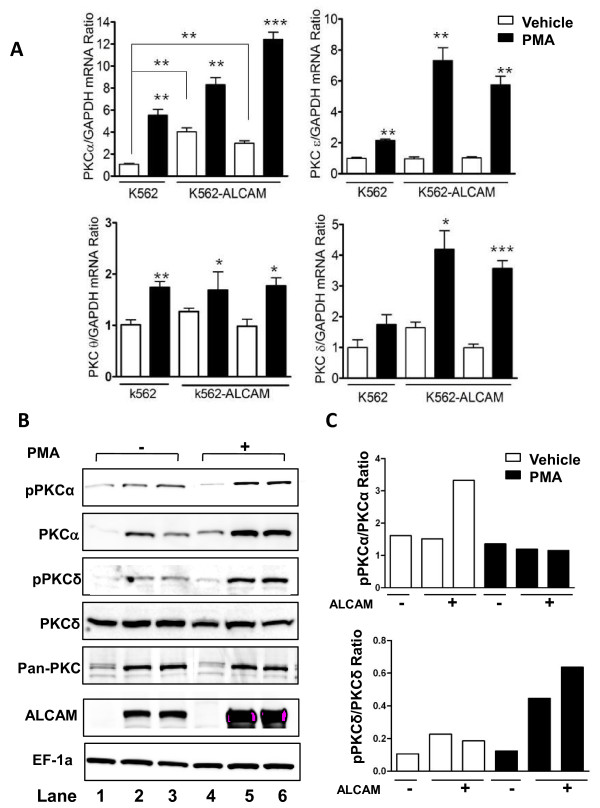
**ALCAM alters expression of PKC in K562 cells**. A) Quantification of PKC mRNA level in K562-ALCAM (#1, #2) and K562 (#15) clones at steady-state (vehicle) and following treatment with 20 nM PMA for 48 h. B) Western blot analysis showing expression of the indicated proteins in control K562 clone #15 (lanes 1, 4), ALCAM-expressing clones #1(lane 2, 5) and #2 (lanes 3, 6) at steady-state (-) and following treatment with PMA (+). C) Quantification of PKCα and PKCδ activation.

To analyze the PKC mechanisms involved in ALCAM-mediated cell clustering, we examined expression of PKC isoforms by qRT-PCR. There was significantly increased expression of PKC-α, but not of PKC-δ, -ε or -θ in these cells at baseline. The level of PKC-α mRNA was 3-fold higher in K562-ALCAM clones than in control cells (Figure 5A). The pan-PKC activator PMA significantly increased mRNA expression of three isoforms (α, ε and θ) in both K562-ALCAM and the control K562-GFP cells (Figure 5A). PKC-δ mRNA expression increased significantly in the K562-ALCAM clones, but not in control cells. Next, we examined steady-state and PMA-induced levels and activation of PKC-α and PKC-δ. The level of PKC-α protein was increased in K562-ALCAM clones, at steady-state and following PMA treatment, however PMA did not increase activation of PKC-α (Figure 5B, C). On the contrary, PMA activated PKC-δ in the K562-ALCAM clones, while the activation status in control cells remained unchanged (Figure 5B, C).

### Altered erythromegakaryocytic gene expression in K562-ALCAM clones

**Figure 6:**
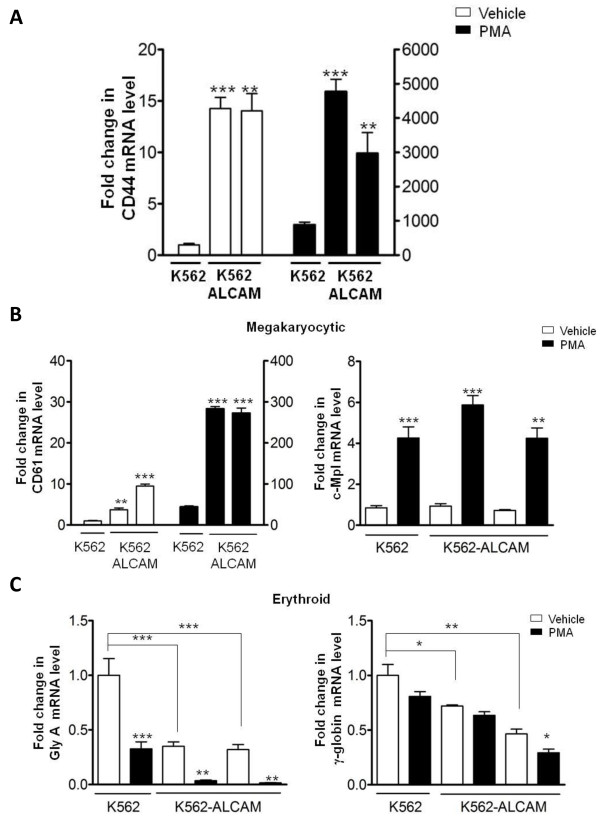
**Profile of PMA-induced gene expression in K562 cells is altered by ALCAM**. Quantification of mRNA for; A) PKC-inducible gene CD44, B) megakaryocytic genes, CD61 and c-Mpl, and C) erythroid genes, glycophorin A and γ-globulin, in K562-ALCAM (#1, #2) and K562 (#15) at steady-state and following treatment with 20 nM PMA for 48 h.

K562 is an established model for megakaryocytic differentiation in a process that is PKC-ε dependent [22,23]. Thus our data showing that ectopic expression of ALCAM in K562 cells increased expression and activation of PKC-α, suggested that the normal megakaryocytic pathway may be altered in these clones. Transcripts for the PMA-inducible gene CD44 was greater than 10-fold higher in the K562-ALCAM clones compared to control K562 cells at baseline, while PMA increased its expression 3 to 5-fold higher in K562-ALCAM clones compared to control K562 cells (Figure 6A). With respect to hematopoietic genes, transcripts for the megakaryocytic marker CD61 was 3-10 fold higher in K562-ALCAM clones in the absence of PMA although this was not matched by a comparable increase in c-Mpl expression (Figure 6B). The level of mRNA for the erythroid genes glycophorin A and γ-globin was significantly reduced in K562-ALCAM clones compared to K652 control at baseline (Figure 6C). As expected PMA increased mRNA level of megakaryocytic genes and concomitantly decreased erythroid gene expression in the various K562 clones (Figure 6B, C). In agreement with our findings at steady-state, c-Mpl expression in K562-ALCAM clones did not increase beyond the level of control K562 cells following induction with PMA. Discordant expression of CD61 and c-Mpl reinforced the notion that megakaryocytic differentiation in the K562-ALCAM clones was likely dysregulated.

### Apoptosis of K562-ALCAM clones undergoing megakaryocytic differentiation

**Figure 7:**
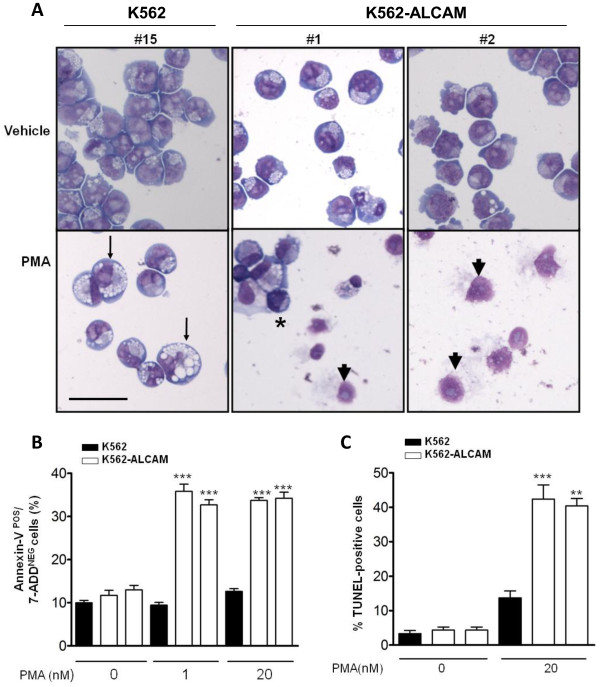
**ALCAM promotes apoptosis of K562 cells treated with PMA**. A) Photomicrographs of control K562 (#15) and K562-ALCAM clones (#1, #2) stained with May-Grunwald Giemsa, at steady-state (upper panel) or following treatment with 20 nM PMA (lower panel) for 48 hours. PMA caused enlargement and extensive vacuolation in K562 cells (arrow) and nuclear fragments in K562-ALCAM cells (arrow head). B) Apoptosis as determined by the expression of Annexin V in K562 and K562-ALCAM cells at steady-state and following treatment with PMA for 24 hours. C) Apoptosis as determined by the TUNEL assay in K562 and K562-ALCAM cells at steady-state and following treatment with PMA for 48 hours. Scale bar: 50 μm.

K562 cells treated with PMA are well known to display multiple characteristic of megakaryocytic differentiation. To further investigate the impact of ALCAM on K562 cells, we examined cells treated with PMA for the morphological modifications that characterize megakaryocytic differentiation. Cells stained with May-Grunwald-Giemsa revealed that control K562 cells displayed multiple phenotypes consistent with megakaryocytic differentiation, including cell enlargement and extensive vacuolation (Figure 7A, lower panel, arrow). On the contrary, cultures of K562-ALCAM treated with PMA were predominated by amorphous nuclear remnants devoid of recognizable cytoplasm (arrow heads). Other cell types in the K562-ALCAM cultures were markedly smaller with condensed chromatin, and fragmented nucleus consistent with apoptosis (Figure 7A, asterisk). Apoptosis in K562-ALCAM clones was confirmed first, by determining the proportion of cells at the early stage of this process, as defined by surface expression of annexin V. At baseline, the proportion of annexin V-positive cells was similar (10%) in cultures of K562-ALCAM and K562 clones. However, treatment of cultures with PMA for 24 hours increased annexin V-positive cells exclusively among K562-ALCAM cells by 3-fold (Figure 7B). Finally, vehicle and PMA-treated cultures were examined for degradation of DNA using the TUNEL assay. At baseline there was no difference in the percentage of TUNEL-positive cells in either cell type, or in cells treated with DMSO (Figure 7C and data not shown). Treatment with 20 nM PMA for 48 hours increased the percentage of TUNEL-positive cells by an average 4-fold higher in K562-ALCAM compared to K562 cultures.

## Discussion

We report that ALCAM is selectively down-regulated in erythromegakaryocytic progenitor cell lines, and that this phenomenon is essential for survival of K562 cells induced to differentiate towards the megakaryocytic lineage. Our findings suggest that erythromegakaryocytic progenitors that escape ALCAM down regulation may contribute to megakaryocytopenia.

ALCAM was originally thought to be a stage-specific molecule rather than a lineage-specific marker, based on observations that its expression was lost as avian hematopoietic progenitor cells proliferated and differentiated into mature colonies of diverse lineages [24]. More recently, it has become clear that ALCAM is expressed by a diversity of mature leukocytes however its lineage identity remained undefined. Data presented in this study show that ALCAM expression is turned off exclusively in progenitor cell lines of the erythromegakaryocytic lineage (Figure 1). Taken together, this suggests that a factor controlling erythromegakaryopoiesis silences ALCAM gene expression. GATA-1 is an excellent candidate for this role given its dominant influence on the survival and differentiation of erythroid and megakaryocytic progenitors [25,26]. Moreover, GATA-1 acts as a gene silencer [27,28] and a lineage-determining factor that suppresses myelomonocytic gene expression while promoting erythroid and megakaryocytic lineage commitment [29]. In support of this paradigm, we found that GATA-1 is abundantly expressed in the K562 and MEG-01 cells used in this study, which lack ALCAM expression, while it is absent in the monocytic cells that abundantly express ALCAM (data not shown). More specifically, we found that there is a GATA-1 *cis *element in the ALCAM promoter that inhibits promoter activity, and is bound by GATA-1 *in vivo*, thus providing additional data to support this general paradigm.

As expected, anti-ALCAM antibody inhibited clustering of K562-ALCAM clones, confirming that this phenomenon is dependent on ALCAM adhesion. Corbel *et al. *[18] used a similar approach to reveal the requirement for ALCAM adhesion in the growth of macrophage, granulocyte and granulocyte/macrophage progenitors. However, anti-ALCAM antibody had no impact on the formation of thromboblast/erythroblast clusters, or growth of erythroid colonies derived from BFU-progenitors [18]. This is in agreement with our findings in wild-type K562 cells grown in media containing anti-ALCAM antibody (data not shown). We have found that the amount of ALCAM mRNA and protein expressed in the human erythroleukemia cell line HEL is virtually undetectable, similar to what is reported here for K562 and MEG-01 (data not shown). While we cannot exclude the possibility that ALCAM is expressed by immature erythromegakaryocytic precursors, ALCAM silencing in mature precursors would facilitate their segregation from their myeloid counterparts that continue to express ALCAM. In this context apoptosis would be an effective mechanism to ensure lineage fidelity among erythromegakaryocytic precursors that continue to express ALCAM, as demonstrated by this study (Figure 7). That ALCAM expression is pro-apoptotic is contrary to its protective role against apoptosis in breast cancer cells [30]. This apparent discordance is consistent however with the notion that ALCAM is at the crossroads of opposing phenotypes such as adhesion and migration [31]. ALCAM may therefore be involved in influencing the balance between survival and apoptosis, at least, in the hematopoietic system.

Megakaryocytic differentiation is orchestrated by multiple signaling pathways including activation of PKCε in both CD34 hematopoietic stem cells and in K562 cells [32]. The major trigger for this event in K562 cells is PMA, which activates multiple PKC isoforms. Ectopic expression of ALCAM in K562 cells activated PKC, with functional downstream effects on cell aggregation and gene expression, in the absence of an external agonist (Figure 3, 4 and 5). PKC isoforms have distinctly unique impacts on survival and apoptosis, with the conventional isoforms, particularly PKC-α and PKC-β being pro-survival [33,34], the novel isoform, PKC-δ is primarily pro-apoptotic [35,36], whereas PKC-ε suppress apoptosis in most studies [37,38]. Enhanced expression of PKC-α in K562-ALCAM clones provided an early indication of a potentially different signaling pattern in these clones. PKC-δ was markedly activated without a comparable activation of the pro-survival PKC-α (Figure 5B, C). This pattern of activation may explain the increased apoptosis of K562-ALCAM clones in response to a signal that normally promotes differentiation towards the megakaryocytic lineage.

## Conclusions

Our data suggest, for the first time, that silencing of ALCAM at the bi-potential erythromegakaryocytic stage may be crucial for cell survival during megakaryocytic differentiation.

## Methods

### Chemicals and antibodies

Phorbol-12-myristate-13-acetate (PMA), Chelerythrine chloride and lipopolysaccharide were purchased from Sigma (St Louis, MO). Monoclonal anti-ALCAM antibody were purchased from Novocatra Laboratories Ltd (Novocatra, Newcastle, UK), anti-PKC isotype-specific antibodies, anti-EF-1α from Upstate (Charlottesville, VA), anti-GATA-1 antibodies and goat anti-mouse IgG-HRP from Santa Cruz Biotechnology (Santa Cruz, CA). Generation of rabbit anti-rat ALCAM antibody BRI-1 has previously been described as anti-HB2 [39,40]. BRI-1 antiserum was purified on a protein-A column (ImmunoPure Plus High Capacity, Pierce Biotechnology, Inc. Rockford, IL).

### Cell culture

K562 and HL-60 cells were cultured in Iscove's Modified Dulbecco's Medium (IMDM) (Invitrogen, Carlsbad, CA), supplemented with 10% fetal bovine serum (FBS) and 1% penicillin and streptomycin sulfate. THP-1, Jurkat, U-937 and MEG-01 cells were cultured in RPMI 1640 medium (ATCC, Manassas, VA) supplemented with 10% FBS. Cells were incubated at 37°C in a humidified chamber with 5% CO_2_. For cell cluster formation analysis, single cell suspensions of K562-ALCAM (2 × 10^5^) were seeded in 24-well plates. Affinity purified BRI-1 antibody (10 μg/ml) or non-immune IgG or chelerythrine chloride (10 μM) was added to cultures and examined 24 hours later. For disaggregation studies, K562-ALCAM cells (2 × 10^5^) were allowed to form large aggregates over a 24-hour period in 24-well plates, and then the cultures were gently treated with BRI-1 antibody (10 μg/ml) or a concentration range (5-20 μM) of chelerythrine chloride. Cultures were photographed using Olympus IX50 microscope with digital image (Olympus Optical Co. Ltd. Japan).

### Western blot analysis

Cell lysates prepared with ice-cold Cell Lysis Buffer (Cell Signaling Technology, Beverly, MA) containing 1% triton X-100 (v/v) and supplemented with 1% protease inhibitor cocktail (Roche, Indianapolis, IN) was clarified by centrifugation at 13,000 rpm for 15 min at 4°C, and soluble cell fractions harvested. Protein content in cell lysates was measured using a Lowry protein assay (Sigma, St Louis, MO). Lysates were combined with buffer (Sigma, St Louis), boiled for 2 min and resolved by electrophoresis on a 10% polyacrylamide gel. Samples were blotted unto nitrocellulose membranes, probed with antibodies. The protein bands were identified by chemiluminescence (Fujifilm, Japan).

### Quantitative RT-PCR

Total RNA was extracted from cultured cells using RNeasy Mini Kit (Qiagen, Valencia, CA), and converted to cDNA using SuperScript RT II (Invitrogen,). Quantitative RT-PCR was performed using gene-specific primers (Table 1), in reactions containing SYBR Green PCR master mixture (Applied Biosystems, Foster City, CA), on a StepOnePlus analyzer (Applied Biosystems). Relative expression level of target genes was normalized using GAPDH mRNA.

**Table 1:** Sequence of Primers and DNA probes

ALCAM Luc reporter constructs	*Forward*	*Reverse*
-1200	5'-AAATCACCGCTTAACTCAAAG-3'	*5'-CCTCCTCCTTCTTGGTGG-3'*
-1000	5'-CCGCGCTTCAACCACCTGCT-3'	
-800	*5'-CAGAAAGTGTTAGTCCCAGG-3'*	
-650	*5'-CCGCGCTTCAACCACCTGCT-3'*	
-400	*5'-CCGCCTCCTGCGAGTCCTTC-3'*	
-200	*5'-GTTGACCGGGAGGGAGGAGG-3'*	
**EMSA probes**	Sense strand	
- 850 GATA-1 wt	5'-GCCAGAGGCCTTATCACTGG-3'	
- 850 GATA-1 mt	5'-GCCAGAGGCCTGCTCACTGG-3'	

**ChIP assay**	*Forward*	*Reverse*
GATA-1	5'-GGGTGGAGGGAGAGGGCAGTC-3'	5'-AAGCAAGCACAGGAGCCGCGC-3'

**RT-PCR**	*Forward*	*Reverse*
ALCAM	5'-AGTCTT CAT TATCAGGATGC-3'	5'-GGGATCAGTTTTCTTTGTCA-3'
γ-globin	5'-ACTCGCTTCTGGAACGTCTGA-3'	5'-GTATCTGGAGGACAGGGCACT-3'
Glycophorin A	5'-GGAATTCCAGCTCATGATCTCAGGATG-3'	5'-TCCACATTTGGTTTGGTGAACAGAT3'
Cmpl	5'-GCACTGTGATGCTTTATGCAAC-3'	5'-TGAACGGTTTAGAGGATGAGGA-3'
CD44	5'-GATCCACCCCAATTCCATCTGTGC-3'	5'-AACCGCGAGAATCAAAGCCAAGGC-3'
CD61	5'-TATAGCATTGGACGGAAGGC-3'	5'-GACCTCATTGTTGAGGCAGG-3'
GAPDH	5'-ATGGGGAAGGTGAAGGTCGG-3'	5'-GACGGTGCCATGGAATTTGC-3'
PKCα	5'-CGAGGAAGGAAACATGGAACTCAG-3'	5'-CCTGTCGGCAAGCATCACCTTT-3'
PKCε	5'-TCAATGGCCTTCTTAAGATCAAAA-3'	5'-CCTGAGAGATCGATGATCACATAC-3'
PKCθ	5'-CTCGTCAAAGAGTATGTCGAATCA-3'	5'-AATTCATTCAGTCCTTTGTGTCACTC-3'
PKCδ	5'-GCATCGCCTTCAACTCCTATGAGCT-3'	5'-ACACACCCACGGTCACCTCAGA-3'

### Stable K562 cells expressing ALCAM

We have previously described the cloning of rat ALCAM-GFP [19]. Super coiled plasmid DNA (2.5 μg) of ALCAM-GFP or GFP was transfected into log-phase growing K562 cells using lipofectamine 2000 (Invitrogen). Stable lines were selected with G418 (1200 μg/ml) for 20 days. Expression of ALCAM-GFP and GFP was examined by epifluorescence (Nikon TE2000, Nikon Instruments Inc., Melville, NY).

### Reporter constructs and Site-directed mutagenesis

Human genomic DNA was amplified by PCR using a common reverse primer complimentary to DNA sequence 60 base pairs (bp) upstream of the translation start site in the ALCAM gene, and various forward primers truncated at -1200, -1000, -800, -650, -400 and -200 (Table 1). These DNA fragments were then cloned into a promoter-less luciferase vector pGL3B (Promega, Madison, WI) via *Kpn *I and *Sma *I cleavage sites. The ALCAM-luciferase vectors (p1200ALCAMLuc, p1000ALCAMLuc, p800ALCAMLuc, p650ALCAMLuc, p400ALCAMLuc and p200ALCAMLuc) were generated and verified by sequence analysis. Two bp substitutions were generated in the GATA-1 binding site at -850 (CCTTATCACT→CCTGCTCACT) by site-directed mutagenesis (QuikChange, Stratagene, La Jolla, CA), and verified by DNA sequencing.

### Reporter gene assays

Cells (2 × 10^5^) were seeded in 24-well tissue culture plates and co-transfected with ALCAM promoter luciferase plasmids (800 ng) and pcDNA3.1/His/LacZ (100 ng) (Invitrogen) plasmid DNA using lipofectamine 2000. Twenty-four hours after transfection, cell lysates were prepared and the activities of luciferase (Firefly-Luciferase Reporter Assay System, Promega ) and β-galactosidase (Galacto-Star system, Applied Biosystems) were determined using the Veritas Luminometer (Turner Biosystems, Sunnyvale, CA). Luciferase activity was normalized to the activity of β-galactosidase, and the relative luciferase activity for test constructs calculated by assigning the normalized luciferase activity of the promoter-less pGL3 construct as 1.0.

### Electrophoretic mobility shift assay (EMSA)

*In vitro *protein-DNA interaction was examined using the LightShift Chemiluminescence electrophoretic mobility shift assay (EMSA) kit (Pierce, Rockford, IL). ALCAM-specific EMSA DNA probes were synthesized, gel purified and biotin labeled (Table 1) (Integrated DNA Technologies, Coralville, IA). Nuclear extract (4 μg) was combined with biotin-labeled DNA probes in binding buffer containing 50 μg/ml poly(dI-dC). Molar excess (50-200-fold) of unlabelled DNA probe or anti-GATA-1 antibody (2 μg) (Santa Cruz Biotechnology) was added to the binding reaction in competition experiments. Products of the binding reaction were resolved in 6% DNA retardation gel (Invitrogen), transferred to a nylon membrane and biotin-labeled complexes detected by chemiluminescence (Fujifilm LAS-1000 imaging system, Valhalla, NY).

### Chromatin immunoprecipitation assay (ChIP)

For ChIP assay, protein-DNA cross-linking was performed by fixing 40 million cells with 1% formaldehyde. Nuclei were sonicated on ice in shearing buffer (ChIP-IT, Active Motif, Carlsbad, CA) to obtain chromatin fragments of 1000-100 base pairs, which were pre-cleared with protein G beads (Salmon sperm DNA/Protein G agarose). Alternatively, pre-cleared chromatin was incubated with anti-GATA-1 (Santa Cruz Biotechnology), anti-GAPD or non-immune IgG. Immune complexes were precipitated with protein G beads, and the eluate reversed cross-linked in 190 mM NaCl. DNA was purified by centrifugation on a mini-column and amplified by PCR with specific ALCAM primers (Table 1). PCR products were resolved by electrophoresis in 2% agarose gels, visualized by ultraviolet illumination and digital images recorded using GeneFlash gel documentation system (SynGene, Frederick, MD).

### Apoptosis and TUNEL assays

Cells (1 × 10^6^) were plated in 6-well plates and incubated with PMA (20 nM) or vehicle (DMSO). Twenty-four hours later, cells were stained with phycoerythrin (PE) labeled annexin V and 7-Amino-actinomycin (7-ADD), using the Apoptosis Detection kit (BD Biosciences, San Jose, CA). Cells were then analyzed by flow cytometry (FACScan Advantage, BD Biosciences) and data from 10,000 events collected for analysis. For the TUNEL (terminal deoxynucleotidyl transferase-mediated deoxyuridine triphosphate nick end labeling) assay, cells (1 × 10^6^) were plated in 6-well plates, and treated for 48 hours with PMA (20 nM) or vehicle (DMSO). Cells were fixed using freshly diluted 1% paraformaldehyde in PBS for 10 minutes at room temp. Cell monolayers were prepared by cytospin on a silanized microscope slide, apoptotic cells were identified using the in situ ApopTag Peroxidase kit (Chemicon International, Temecula, CA). Methyl green solution (0.5%) was used to counter-stain the slides. Apoptotic cells were counted in 10 random fields using 20× microscope objective and the percentage TUNEL-positive cells calculated.

### Statistics

Data were analyzed using GraphPad Prism software (version 5). Student's *t-*test was used to measure differences in samples of two groups. Probability of less than 0.05 was considered statistically significant. Levels of significance are: p < 0.05 (*), p < 0.01 (**) and p < 0.001 (***).

## List of abbreviations used

ALCAM: activated leukocyte cell adhesion molecule; RACE: rapid amplification of cDNA ends; EMSA: Electrophoretic mobility shift assay; CHiP: Chromatin immunoprecipitation assay.

## Authors' contributions

FT performed experiments and prepared the manuscript. SG, FM and RT performed experiments, JAW provided ALCAM null mice and SFOA conceived of the study, interpreted data and wrote the manuscript. All authors read and approved the final manuscript.
